# The Impact of Data Control and Delayed Discounting on the Public’s Willingness to Share Different Types of Health Care Data: Empirical Study

**DOI:** 10.2196/66444

**Published:** 2025-01-22

**Authors:** Dongle Wei, Pan Gao, Yunkai Zhai

**Affiliations:** 1 School of Management, Zhengzhou University, Zhengzhou, China

**Keywords:** health data control, delay discounting rate, mental accounting, health data, data sharing, willingness, patient-generated data, clinical medical data, disease prevention, precision medicine, health care, portability, accountability, app, web-based survey, data security, data privacy, mobile phone

## Abstract

**Background:**

Health data typically include patient-generated data and clinical medical data. Different types of data contribute to disease prevention, precision medicine, and the overall improvement of health care. With the introduction of regulations such as the Health Insurance Portability and Accountability Act (HIPAA), individuals play a key role in the sharing and application of personal health data.

**Objective:**

This study aims to explore the impact of different types of health data on users’ willingness to share. Additionally, it analyzes the effect of data control and delay discounting rate on this process.

**Methods:**

The results of a web-based survey were analyzed to examine individuals’ perceptions of sharing different types of health data and how data control and delay discounting rates influenced their decisions. We recruited participants for our study through the web-based platform “Wenjuanxing.” After screening, we obtained 257 valid responses. Regression analysis was used to investigate the impact of data control, delayed discounting, and mental accounting on the public’s willingness to share different types of health care data.

**Results:**

Our findings indicate that the type of health data does not significantly affect the perceived benefits of data sharing. Instead, it negatively influences willingness to share by indirectly affecting data acquisition costs and perceived risks. Our results also show that data control reduces the perceived risks associated with sharing, while higher delay discounting rates lead to an overestimation of data acquisition costs and perceived risks.

**Conclusions:**

Individuals’ willingness to share data is primarily influenced by costs. To promote the acquisition and development of personal health data, stakeholders should strengthen individuals’ control over their data or provide direct short-term incentives.

## Introduction

### Background

Health data encompass information related to medical diagnosis, treatment, prevention, and health risks [1,2]. This includes patient-generated data (PGD) and clinical medical data. PGD refer to health-related data created, recorded, or collected by patients [3,4] often through automatic tracking devices like smartphones and wearables as well as manual logs and surveys. Conversely, clinical medical data are derived from clinical research, medical diagnostic records, and health insurance company records. Using public health data can significantly reduce operational costs for health care providers and enhance the quality of services provided [5,6]. Sharing health data allows physicians to gain detailed insights into patients’ conditions, facilitating the development of personalized treatment plans and enabling the exploration of new treatments for diseases such as cancer [7]. For the public, health data sharing and interaction offer substantial benefits, including the reduction of unnecessary hospitalizations, the avoidance of duplicate medical tests, and the prevention of adverse drug events [8,9]. Furthermore, the application of public health data aids policy makers in formulating equitable health care policies and improving public health management at a societal level [1,10]. The National Institutes of Health recently announced the “All of Us” initiative, aiming to collect data from 1 million or more patients, including electronic health records, medical imaging, sociobehavioral data, and environmental data.

Despite the potential advantages of data use, public willingness to share health data is shifting [11]. Sharing health data involves intertemporal decision-making, where individuals must balance benefits and costs over time [12,13]. The costs may include psychological, financial, or other nonfinancial costs, while the benefits relate to potential future advantages such as reduced disease incidence and improved personal health management. The public’s delay discounting rate significantly impacts these intertemporal decisions. Individuals with a high delay discounting rate prefer immediate rewards, whereas those with a low rate prioritize long-term benefits [14,15]. Unlike other health behaviors, sharing health data involves both health benefits and the risk of privacy breaches. Different types of health data carry varying levels of benefits and risks. Clinical medical data, for example, present higher privacy concerns and greater application value compared to PGD. Health data contain vast amounts of sensitive personal information, and any breach can lead to significant social and economic losses for individuals, thus decreasing their willingness to share such data [16,17]. Additionally, social stigmatization is a significant barrier to sharing sensitive health data with numerous health care organizations [18-20]. For instance, a data breach in a health information exchange (HIE) could reveal genetic diseases, causing distress for affected families.

Simultaneously, the unique nature of health data has made the issue of related rights a significant topic of discussion within the health care industry and academia. A key factor contributing to the public’s reluctance to share health data is their lack of control over it [19,21]. The public cannot ascertain how other organizations or individuals will use their health data once it is acquired nor can they know whether their data will be transmitted to third parties [20]. Many people fear that their personal medical information could be used for marketing purposes without their authorization, posing a significant privacy risk [20]. Reports indicate that many American adults are highly anxious about how their health information is accessed and used [22]. Currently, it is widely accepted that other institutions or individuals must obtain the data owner’s consent before acquiring and using health data in accordance with relevant laws. For instance, the health care industry has established policies and regulations such as the Health Insurance Portability and Accountability Act (HIPAA) and the General Data Protection Regulation. Therefore, understanding public willingness to share health data is crucial for the future development of public health initiatives. Our study, based on the theory of mental accounting, explores how different types of health data impact the public’s willingness to share their data through perceived benefits and costs. Additionally, it analyzes the mechanisms by which the public’s delay discounting rate and health data control influence their willingness to share health data.

### Related Work

With the increasing prevalence of patient-centered design (PCD), the role and authority of the public in the sharing and interaction of health data have garnered significant attention. Existing research has analyzed the factors influencing health data sharing from various perspectives. For example, van Panhuis et al [23] conducted a systematic review of potential barriers to public health data sharing, identifying 20 potential barriers and categorizing them into 6 types: technical, motivational, economic, political, legal, and ethical. Sun et al [24], from the perspective of satisfaction, analyzed the factors influencing users of web-based health communities in disclosing their health information. Demographic characteristics such as participant age, region of residence, country or region of residence, and education level have a certain impact on the willingness to share personal health data [11]. Additionally, the public’s ability to access, understand, and use health data directly influences their willingness to share health data [7].

Moreover, the privacy of health data is a crucial concern for patients, and preferences vary depending on the type of health data and the characteristics of the patients involved [21,25]. Watson et al [19], through semistructured interviews with patients, identified data type as a significant factor influencing patients’ willingness to share personal health data. Patients’ preferences for sharing PGD vary: some consistently share their data to keep their clinicians informed [3], while others refuse to share due to privacy concerns or fear of judgment [18,26]. Some public limit sharing their PGD to only trusted clinicians [27]. Moreover, research indicates that the public tends to avoid sharing sensitive health data [28]. Kim et al [29] collected participants’ exercise and sleep data through experiments and used questionnaires to analyze their views on sharing daily health data. Kirkham et al [18] used ordinal logistic regression to study the impact of demographic factors, clinical service experience, and primary mental illness on the willingness to share mental health data, comparing it with physical health data. Seltzer et al [30] analyzed patients’ willingness to share different types of data. The results showed that 50% of patients agreed to share at least 1 type of digital data, and 78% agreed to donate at least 1 type of data posthumously, with the highest percentages for electronic medical record, wearable device data, and Google search histories. Kim et al [31] and Mello et al [32], respectively, analyzed the types of institutions to which participants are more inclined to share their medical records and biospecimens. However, there is limited research that focuses on the impact of both PGD and clinical medical data on the public’s willingness to share data as well as the underlying mechanisms of these effects.

Existing research increasingly emphasizes the rights of patients regarding their health data. Scholars have studied patients’ control over health data from ethical [33], legal [34,35], managerial [36], and other perspectives. Currently, the entire process of HIE is managed by health care institutions for the interaction. In this process, patients, as the owners and beneficiaries of health information, often have their rights overlooked [37]. According to Hunter [38], as the quantity and scope of collected personal health data increase, the greatest demand is for transparency in their use. Kish and Topol [39] point out that to fully realize the benefits of digital health care, we need not only to find a shared home for personal health data but also to empower individuals to own them. PCD increases patients’ willingness to share health data with others [40]. Esmaeilzadeh [41], drawing on utility theory, explores the factors influencing patients’ adoption of HIE from the perspectives of perceived value and perceived risk. The public expects to know the purpose of sharing their health data and desires control over data circulation [28]. Karway et al [42] found in their research that patients prefer to share their health data when they can control it themselves. The majority (87%) also reported feeling more comfortable sharing their data when they know its purpose. Sanderson et al [43] examined the public’s willingness to share their biological data under different consent scenarios. In contrast, our study focuses on the public’s evaluation of the current situation and examines the interaction effect between delay discounting and data control.

While existing research has extensively studied the factors influencing the public’s willingness to share health data, much of it has been conducted through structured interviews, literature analysis, and similar methods. The mechanisms through which types of health data and the public’s control over data affect their willingness to share remain unclear. Additionally, as intertemporal decisions, the public’s preference for delay discounting rate directly impacts their data-sharing behavior, but there has been no research analyzing its underlying mechanisms. Therefore, our study poses the following research questions: How do different types of health data affect the public’s willingness to share through benefits and costs? What is the role of the public’s control over their health data and delay discounting rate in health data sharing?

### Research Model and Hypotheses

The benefits and costs inherent in different types of health data vary. Our study categorizes health data into PGD and clinical medical data. Compared to clinical medical data, PGD entail lower sensitivity and fewer legal compliance requirements. Clinical medical data, comprising medical diagnostic records, clinical research data, and health insurance company records, are highly sensitive and private, necessitating strict adherence to regulations such as HIPAA. Consequently, patients are more inclined to share PGD rather than clinical medical data due to the relatively less sensitive nature of PGD information. In contrast, clinical medical data involve highly sensitive personal health information, raising concerns about privacy breaches and data misuse. Furthermore, PGD acquisition costs are lower, as it is obtained through various wearable smart devices and the internet. However, the self-reporting nature and lower accuracy of PGD may diminish its value in medical research and clinical applications. In contrast, clinical medical data possess high precision and professional recording, making it directly applicable to medical research, clinical trials, and precision medicine, thus yielding higher sharing benefits. However, acquiring clinical medical data requires involvement from medical institutions or insurance companies, necessitating strict permission management and incurring potential costs and time expenses. Hence, we hypothesize the following:

- Hypothesis 1: The public exhibits a higher willingness to share PGD compared to clinical medical data.
- Hypothesis 2: The relationship between the type of health data and willingness to share data is mediated by perceived benefits (hypothesis 2a), perceived risks (hypothesis 2b), and data acquisition costs (hypothesis 2c).

The concept of mental accounting, introduced by Thaler [44], is a key area in behavioral decision-making. Tversky and Kahneman [45] further defined it as the cognitive processes of categorizing, encoding, evaluating, and budgeting outcomes. It essentially involves assessing gains and losses during decision-making [46]. Since its introduction, mental accounting theory has found extensive applications in behavioral economics, consumer behavior, and decision-making [47-49]. Additionally, Hossain [50] explored how individual cognition influences mental accounting and its application to different product types.

Moreover, mental accounting influences individuals’ engagement in health data-sharing behavior. In the context of health data sharing, individuals tend to view the potential benefits of sharing as part of the “benefit account.” The greater the perceived benefits, the more likely they are to share health data. Meanwhile, the potential risks of privacy leakage are classified under the “loss account.” The stronger the perception of risk, the lower the individual’s willingness to share data. Furthermore, the theory of mental accounting also highlights the impact of sunk costs—time and effort already invested are seen as irrecoverable losses, which, in turn, reduce the individual’s willingness to continue sharing data. Hence, we propose the following hypotheses:

- Hypothesis 3: Perceived benefits positively affect the willingness to share health data.
- Hypothesis 4: Perceived risks negatively affect the willingness to share health data.
- Hypothesis 5: Data acquisition costs negatively affect the willingness to share health data.

The notion of health data control pertains to individuals’ rights to manage their health care information. Drawing from the theory of psychological ownership, individuals tend to perceive objects they own as extensions of themselves, influencing their attitudes, motivations, and behaviors [51]. Psychological ownership refers to the feeling individuals have that an object belongs to them, emphasizing their sense of possession [52]. In our study, health data serve as the focus of psychological ownership. Belanger and James [53] discovered that users prefer direct control over personal privacy compared to corporate control. Direct control enhances users’ self-efficacy and willingness to disclose personal information.

Moreover, PCD significantly impacts patients’ adoption of electronic health record systems. PCD ensures that all users within health care institutions can access patient health information with patient confirmation [54]. PCD increases patients’ willingness to share health data [40] and empowers them to manage their information. Greater public control over health data reduces perceived privacy risks and enhances awareness of data-sharing benefits while downplaying associated costs. Thus, we propose the following hypothesis:

- Hypothesis 6: Health data control increases the perceived benefits (hypothesis 6a) and reduces the perceived risks (hypothesis 6b) in data sharing.

Intertemporal decision-making involves balancing and judging between time and gains or losses [55]. It includes choices like immediate gratification versus future enjoyment or the immediate pleasure of smoking versus future health concerns. This process, known as intertemporal choice, reflects the value people assign to payoffs at different times [56]. Most decisions require trading off costs and benefits over time. Delay discounting rate and subjective value are key indicators of intertemporal choice; a higher discounting rate or lower subjective value suggests greater impulsivity and preference for immediate rewards over larger future gains [14,15].

Research has examined intertemporal decision-making in health care. Snider et al [57] studied how intertemporal preferences affect adverse health and financial behaviors using delay discounting rates. Tian et al [58] investigated the role of delay discounting rate in adolescent internet gaming addiction. Studies also show that altering intertemporal preferences can improve health behaviors. Interventions that reduce delay discounting rate or enhance future time orientation have been shown to promote better health behaviors [59]. Individuals with high delay discounting rates prioritize immediate gratification, undervaluing health data-sharing benefits and focusing on privacy risks. Conversely, those with lower rates prioritize long-term benefits, emphasizing data-sharing benefits and overlooking privacy risks and data acquisition costs. Therefore, we hypothesize the following:

- Hypothesis 7: Delay discounting rate reduces perceived benefit (hypothesis 7a), increasing perceived risk (hypothesis 7b) and data acquisition costs (hypothesis 7c).

**Figure 1.**
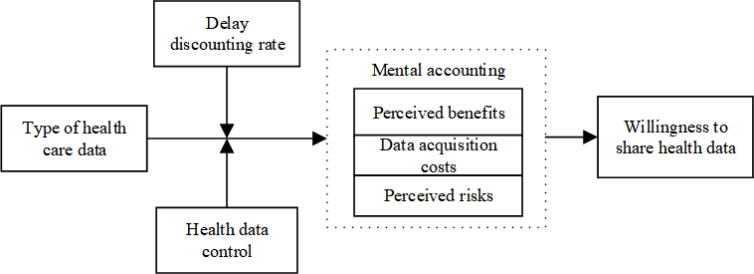
Theoretical model.

Consequently, we propose a moderated mediation model (Figure 1) that is mediated by 3 factors in mental accounting, using health data control and delay discounting rate as moderators.

## Methods

### Participants and Procedure

This study was conducted through the web-based questionnaire platform “Wenjuanxing.” After excluding invalid and incomplete questionnaires, a total of 257 valid responses were collected. To ensure the diversity and representativeness of the sample, recruitment information was posted on the platform. The recruitment notice clearly stated that participants must be between the ages of 20 and 69 years, with no specific educational background requirements, aiming to include individuals from various educational backgrounds. Prior to completing the questionnaire, participants were required to confirm their voluntary participation and understanding of all relevant research information, including the research objectives, expected time commitment, and potential risks involved.

At the beginning of the questionnaire, all participants were presented with a detailed informed consent form, which included a statement of voluntary participation, an explanation of data collection and use, a commitment to protecting personal privacy, and assurances of data anonymity. Participants were required to read this consent form carefully and check a consent box before proceeding with the questionnaire. All data collected were strictly for academic research purposes. Upon completion of the questionnaire, participants were rewarded with US $0.7‐$1.4 based on the completeness and quality of their responses. This incentive was designed to encourage careful and thoughtful participation. To ensure the reliability of the research results, all data were anonymized to prevent any personal identifying information (eg, names, contact information, and IP addresses) from being recorded.

The ages of participants ranged from 20 to 69 years. The majority of participants held undergraduate degrees (n=122, 47.5%), followed by master degree holders (n=70, 27.2%), individuals with college degrees or below (n=55, 21.4%), and doctoral students (n=10, 3.9%).

### Ethical Considerations

This study has obtained ethics approval from the institutional review board of the School of Management, Zhengzhou University. In accordance with the regulations of the institutional review board, this research is deemed to meet ethical standards, and a series of measures have been implemented to ensure ethical compliance throughout the study process. Specifically, all participants’ data have been anonymized to ensure the strict protection of their privacy. Throughout the research, no personal identifying information will be linked to the data, and all raw data will be restricted to use by the research team only. Furthermore, participants were fully informed of the research purpose, the manner in which their data would be used, and the commitment to privacy protection, and they voluntarily consented to participate.

### Overview of the Scenarios

In our study, health data sharing refers to sharing one’s own data with health care providers. Health care providers include hospitals, health insurance companies, and medical device companies. The questionnaire consisted of 3 parts. The first part used nominal scales to collect demographic information, including sex, age, and educational level. The second and third parts focused on variables related to the willingness to share PGD and clinical medical data, respectively. Participants respond using a 5-point Likert scale (1=strongly disagree and 5=strongly agree).

For data types, we classify them as PGD and clinical medical data. PGD include, but are not limited to, the following parts: data measured by wearable smart devices or home medical and health devices, data generated by daily activities, and internet health data. Clinical medical data are mainly electronic medical record data, medical image data, laboratory test data, etc. We divide mental accounts into perceived benefits, perceived risks, and data acquisition costs. Perceived benefits are people’s self-perception of the benefits of sharing health care data based on their own expertise and experience. Perceived risk measures people’s assessment of the risk of data leakage in the process of data sharing and the negative impact of data leakage on them. Data acquisition cost, on the other hand, refers to the effort people put into obtaining relevant health care before sharing health care data. The moderating variables in the paper are data control and delay discount, while data control refers to the degree of control people have over their own health care data. Delay discount rates were calculated using the 27-item Monetary Choice Questionnaire [14]. Each question contained an immediate reward option (eg, US $1.5 today) and a delayed reward option (eg, US $3 in 7 days). We simply replace the US dollar ($) with the Chinese yuan (¥) based on World Bank data. We still use Kirby’s method to calculate the discount rate. In the Kirby task, the 27 trails were divided into 3 groups according to the magnitude of the delay reward, and the discount rates corresponding to the 9 trails in each group were ranked from smallest to largest (0.00016-0.250).

## Results

### Demographics

This study includes several demographic factors, such as sex (male and female) and education level among adolescents. Among the 257 participants, 40.9% (n=105) were male, and 59.1% (n=152) were female. Furthermore, among the respondents, 3.1% (n=8) were aged 20 years and younger, 48.6% (n=125) were aged 21‐29 years, 16% (n=41) were aged 30‐39 years, 13.6% (n=35) were aged 40‐49 years, and 18.7% (n=48) were aged 49 years and older. Additionally, 21.4% (n=22) of individuals had education levels below an undergraduate degree, 47.5% (n=122) had undergraduate degrees, 27.2% (n=70) had master’s degrees, and 3.9% (n=10) had doctoral degrees (Table 1).

**Table 1.** Demographic characteristics.

Variable	Values, n (%)
**Sex**	
Male	105 (40.9)
Female	152 (59.1)
**Age (years)**	
<20	8 (3.1)
21‐29	125 (48.6)
30‐39	41 (16)
40‐49	35 (13.6)
>50	48 (18.7)
**Education**	
Below an undergraduate degree	22 (21.4)
Undergraduate degree	122 (47.5)
Master’s degree	70 (27.2)
Doctoral degree	10 (3.9)

### Preliminary Analyses

Table 2 presents the Pearson and point-biserial correlation coefficients of the variables. Data acquisition cost positively correlates with the data type. Perceived risk positively correlates with sex and data type. The delay discounting rate positively correlates with sex and negatively correlates with education. Health data control positively correlates with education and data type. Willingness to share health data negatively correlates with data type, data acquisition cost, perceived risk, and health data control and positively correlates with perceived benefits.

**Table 2.** Pearson and point-biserial correlation coefficients.

Variable	Sex	Age	Education	DT^a^	DC^b^	PB^c^	PR^d^	DR^e^	HC^f^	HS^g^
**Sex**										
*r*	1	0.038	0.083	0.000	0.009	−0.013	0.105	0.104	0.068	−0.037
*P* value	—^h^	.42	.08	>.99	.85	.78	.03	.03	.15	.43
**Age**										
*r*	0.038	1	−0.468	0.000	−0.064	0.038	0.003	0.048	−0.009	0.008
*P* value	.42	—	<.001	>.99	.17	.42	.96	.31	.84	.86
**Education**										
*r*	0.083	−0.468	1	0.000	0.038	0.049	0.010	−0.146	0.100	0.063
*P* value	.08	<.001	—	>.99	.41	.30	.83	.002	.03	.18
**DT**										
*r*	0.000	0.000	0.000	1	0.485	−0.043	0.482	0.000	0.213	−0.354
*P* value	>.99	>.99	>.99	—	<.001	.36	<.001	>.99	<.001	<.001
**DC**										
*r*	0.009	−0.064	0.038	0.485	1	−0.001	0.435	−0.025	0.085	−0.374
*P* value	.85	.17	.41	<.001	—	.98	<.001	.59	.07	<.001
**PB**										
*r*	−0.013	0.038	0.049	−0.043	−0.001	1	0.023	0.065	−0.112	0.306
*P* value	.78	.42	.30	.36	.98	—	.63	.17	.02	<.001
**PR**										
*r*	0.105	0.003	0.010	0.482	0.435	0.023	1	−0.078	0.328	−0.342
*P* value	.03	.96	.83	<.001	<.001	.63	—	.10	<.001	<.001
**DR**										
*r*	0.104	0.048	−0.146	0.000	−0.025	0.065	−0.078	1	−0.035	−0.010
*P* value	.03	.31	.002	>.99	.59	.17	.010	—	.46	.83
**HC**										
*r*	0.068	−0.009	0.100	0.213	0.085	−0.112	0.328	−0.035	1	−0.109
*P* value	.15	.84	.03	<.001	.07	.01	<.001	.46	—	.02
**HS**										
*r*	−0.037	0.008	0.063	−0.354	−0.374	0.306	−0.342	−0.010	−0.109	1
*P* value	.43	.86	.18	<.001	<.001	<.001	<.001	.83	.02	—

a DT: type of health care data.

b DC: data acquisition cost.

c PB: perceived benefit.

d PR: perceived risk.

e DR: delay discounting rate.

f HC: health data control.

g HS: willingness to share health data.

h Not available.

### Hypotheses Testing

Table 3 shows the direct effects of each variable on the impact of health data sharing. The type of health data has a significant and direct effect on willingness to share (β=−0.28, SE=0.10; *P*<.001), supporting hypothesis 1. Perceived benefits positively influence the willingness to share (Table 2) health data (β=0.40, SE=0.06; *P*<.001), supporting hypothesis 3. Additionally, the data acquisition cost and perceived risk have significant negative direct effects on types of health data, supporting hypothesis 4 and hypothesis 5.

**Table 3.** The direct effect of the variable on health data sharing.

Paths	β (SE)	LLCI^a^	ULCI^b^
DT^c^	−0.28 (0.10)	−0.47	−0.10
DC^d^	−0.27 (0.06)	−0.38	−0.16
PR^e^	−0.18 (0.05)	−0.27	−0.08
PB^f^	0.40 (0.06)	0.29	0.51

a LLCI: lower level of confidence interval.

b ULCI: upper level of confidence interval.

c DT: type of health care data.

d DC: data acquisition cost.

e PR: perceived risk.

f PB: perceived benefit.

The results, as summarized in Table 4, indicate that there is no significant relationship between types of health data and perceived benefit (β=−0.06; *P*=.68; *t*_249_=−0.92), suggesting that types of health data do not directly impact perceived benefit. However, it is noteworthy that in the moderated mediation model, participants’ education level has a positive influence on perceived benefit (β=0.11; *P*=.02; *t*_249_=2.24).

**Table 4.** Regression coefficients in the mediation model and moderated mediation model.

	Data acquisition cost				Perceived risk				Perceived benefit			
	Model (1)		Model (2)		Model (1)		Model (2)		Model (1)		Model (2)	
	β value	*t* test (*df*=249)	β value	*t* test (*df*=249)	β value	*t* test (*df*=249)	β value	*t* test (*df*=249)	β value	*t* test (*df*=249)	β value	*t* test (*df*=249)
DT^a^	0.82^b^	11.78	0.27	1.10	0.97^b^	11.73	1.20^b^	4.53	−0.06	−0.92	0.25	1.12
DR^c^	—^d^	—	−0.06^e^	−2.38	—	—	−0.08^f^	−3.05	—	—	0.03	1.17
DT×DR	—	—	0.09^e^	2.65	—	—	0.09^e^	2.10	—	—	0.00	0.17
HC^g^	—	—	−0.08	−1.58	—	—	0.33^b^	5.62	—	—	−0.02	−0.39
DT×HC	—	—	0.10	1.37	—	—	−0.22^e^	−2.57	—	—	−0.11	−1.72
DC^h^	—	—	—	—	—	—	—	—	—	—	—	—
PR^i^	—	—	—	—	—	—	—	—	—	—	—	—
PB^j^	—	—	—	—	—	—	—	—	—	—	—	—
Sex (male=0; female=1)	0.08	0.25	0.30	0.42	0.22^e^	0.08	0.20^e^	2.48	−0.03	−0.50	−0.04	−0.62
Age	−0.04	−1.28	−0.04	−1.27	−0.00	−0.02	−0.01	−0.31	0.05	1.49	0.05	1.65
Education	0.01	0.20	0.00	0.91	0.00	0.02	0.04	−0.70	0.08	1.65	0.11^f^	2.24
Constant	2.88	14.16	3.27	12.86	2.47	10.13	2.05	7.04	3.36	16.66	3.23	12.87
*R* ^2^	0.49	0.49	0.50	0.50	0.49	0.49	0.56	0.56	0.09	0.09	0.18	0.18

a DT: type of health care data.

b *P*<.001.

c DR: delay discounting rate.

d Not available.

e *P*<.05.

f *P*<.01.

g HC: health data control.

h DC: data acquisition cost.

i PR: perceived risk.

j PB: perceived benefit.

Furthermore, the results demonstrate that types of health data positively influence data acquisition costs (β=0.82; *P*<.001; *t*_249_=11.78) and perceived risk (β=0.97; *P*<.001; *t*_249_=11.73). Interestingly, sex also has a significant influence on perceived risk, with female participants perceiving higher risk in data sharing compared to male participants.

These findings suggest that while the types of health data themselves may not directly impact perceived benefit, they do influence both data acquisition cost and perceived risk. Additionally, participants’ education level plays a role in shaping their perceptions of the benefits associated with sharing health data.

Table 5 shows the indirect effects of types of health data on the impact of health data sharing. Both data acquisition cost and perceived risk have significant mediating effects on the relationship between types of health data and willingness to share health data (β=−0.22, SE=0.05; odds ratio [OR] 0.80, 95% CI −0.33 to −0.12 and β=−0.17, SE=0.06; OR 0.84, 95% CI −0.28 to −0.07), supporting hypothesis 2b and hypothesis 2c. However, the mediating effect of perceived benefits on the relationship between types of health data and willingness to share health data is not significant (β=−0.03, SE=0.03; OR 0.97, 95% CI −0.08 to 0.03), hypothesis 2a was not supported.

**Table 5.** The indirect effect of the variable on health data sharing.

Paths	β (SE)	LLCI^a^	ULCI^b^
DT^c^→DC^d^→HS^e^	−0.22 (0.05)	−0.33	−0.12
DT→PR^f^→HS	−0.17 (0.05)	−0.28	−0.07
DT→PB^g^→HS	−0.03 (0.04)	−0.08	0.03

a LLCI: lower level of confidence interval.

b ULCI: upper level of confidence interval.

c DT: type of health care data.

d DC: data acquisition cost.

e HS: willingness to share health data.

f PR: perceived risk.

g PB: perceived benefit.

Meanwhile, the relationship between types of health care and perceived benefits is not moderated by health data control and delay discount rate (β=−0.11; *P*=.13; *t*_249_=−1.72 and β=0.01; *P*=.97; *t*_249_=0.17), hypothesis 6a and hypothesis 7a were not supported. Additionally, Table 6 indicates that the relationship between types of health data and willingness to share health data through the cost of data acquisition and perceived risk is influenced by delay discounting (β=0.09, SE=0.03; *P*=.01 and β=0.09, SE=0.04; *P*=.03), supporting hypothesis 7b and hypothesis 7c. Health data control moderates the relationship between types of health data and willingness to share health data through perceived risk (β=−0.23, SE=0.08; *P*=.004), supporting hypothesis 6b.

**Table 6.** Moderating effect of delay discounting rate and health data control.

Paths	β (SE)	LLCI^a^	ULCI^b^
**DT^c^→DC^d^→HS**^e^ **(DR^f^)**			
DR=mean−1SD	0.65 (0.09)	0.46	0.83
DR=mean	0.81 (0.06)	0.68	0.95
DR=mean＋1SD	0.98 (0.10)	0.79	1.17
**DT→PR^g^→HS (DR)**			
DR=mean−1SD	0.80 (0.11)	0.58	1.02
DR=mean	0.96 (0.08)	0.80	1.12
DR=mean＋1SD	1.14 (0.11)	0.91	1.36
**DT→PR→HS (HC^h^)**			
HC=mean−1SD	1.10 (0.11)	0.88	1.33
HC=mean	0.87 (0.08)	0.71	1.03
HC=mean＋1SD	0.63 (0.12)	0.40	0.86

a LLCI: lower level of confidence interval.

b ULCI: upper level of confidence interval.

c DT: type of health care data.

d DC: data acquisition cost.

e HS: willingness to share health data.

f DR: delay discounting rate.

g PR: perceived risk.

h HC: health data control.

## Discussion

### Overview

With the advent of the digital age, the application and sharing of health data have garnered significant attention. However, issues such as privacy breaches and ambiguous ownership have emerged, resulting in individuals’ reluctance to share their health care information. Scholars have increasingly focused on strategies to enhance public willingness to share or disclose their health data with institutions. Nonetheless, the influence of different data types on individuals and the moderating effects of delay discounting and health data control remain underexplored. This study aims to analyze the impact of different types of health data on individual data sharing through the lens of psychological ownership theory and examine the moderating effects of delay discounting and health data control.

### Principal Findings

Consistent with other studies, our study demonstrates that higher perceived benefits and lower perceived risks are associated with an increased willingness to share health data [24,41]. Building on this foundation, the study also explores the impact of the effort individuals invest in obtaining data on their willingness to share it. The findings indicate a correlation between lower data acquisition costs and a greater willingness to share data. When individuals expend considerable effort to obtain data, they develop a sense of psychological ownership, perceiving the data as something they have acquired at a cost and therefore value more highly. Due to this psychological ownership effect, they become more cautious and conservative about sharing the data, showing reluctance to share it easily with others. As indicated by Karway et al [42], the public is more inclined to share their health data when they can exercise control over it.

Types of health data may not only directly influence the willingness to share data but also indirectly affect it through mental accounting. Our mediation model revealed that types of health data negatively impact individuals’ willingness to share data by increasing data acquisition costs and perceived risks. Obtaining clinical medical data requires more effort compared to everyday health data due to its complexity and sensitivity, which involves more permissions and privacy protection measures during the acquisition process. Additionally, clinical medical data contain more sensitive information, and its leakage may result in more severe consequences [28,30]. Consequently, the public perceives higher risks associated with sharing such data, resulting in a greater reluctance to share it.

However, contrary to our hypothesis, types of health data do not influence the public’s perception of data-sharing benefits. Whether it is PGD or clinical medical data, the public may prioritize whether data sharing can improve the quality of health care services and enhance health management. However, the public cannot ascertain how their data will be used after sharing nor can they accurately perceive the benefits to themselves after data sharing [22,23]. For different types of health data, the differences in the perceived benefits of data sharing by the public may not be significant.

In the moderated mediation model, participants’ educational level has a positive influence on perceived benefits. This indicates that individuals with higher education levels are more inclined to perceive higher benefits from data sharing. Individuals with higher education levels typically possess greater information literacy and understanding, enabling them to recognize better the potential benefits of data sharing in health management, disease prevention, and medical research. Therefore, they have a more positive perception of the benefits of data sharing. Similarly, sex has a positive influence on participants’ perception of data-sharing risks, with female participants perceiving higher risks compared to male participants. This finding is consistent with existing literature, where female participants typically demonstrate higher sensitivity and caution regarding data privacy and security [60,61]. Female participants may exhibit greater sensitivity to data privacy issues due to societal processes emphasizing security concerns or experiences of insecurity in the use and management of health data.

The delay discounting rate has a positive moderating effect on the relationship between types of health data and the effort participants expend in obtaining data as well as their perceived risks. Individuals with a higher delay discounting rate tend to be more impulsive, with a lower valuation of future rewards. This leads them to rely more on immediate costs and benefits rather than long-term gains in decision-making [14,15]. This psychological characteristic may lead them to perceive the effort required to obtain data as part of the decision-making process. Additionally, compared to long-term health management and potential benefits, individuals with a higher delay discounting rate may prioritize immediate security and privacy protection. Consequently, they may overly focus on the immediate risks associated with sharing medical data, such as privacy breaches and data misuse, while overlooking the potential long-term health benefits and medical advancements that data sharing could bring about.

The relationship between types of health data and perceived data-sharing risks is negatively moderated by health data control rights. Individuals with higher health data control rights typically have more resources and means to simplify the data acquisition process, reduce the complexity and difficulty of obtaining data, and enhance their data protection capabilities, thereby mitigating the perceived risks associated with data sharing [42]. Additionally, granting the public more control over their health data increases their engagement in data sharing, thereby promoting it [40]. Enhancing transparency in the data-sharing process can further empower the public with greater control over their health data [38].

The following insights were gained: to enhance participation in data sharing, health care institutions and policy makers should simplify the data authorization and acquisition processes. By reducing complex authorization steps and clarifying the permissions involved in the sharing process, individuals’ psychological burden and practical operational costs during data sharing can be significantly reduced. For example, adopting a unified user authorization platform or a “single consent” mechanism can simplify the public’s decision-making process regarding data sharing. Additionally, users should be clearly informed about the context and purpose of data use, and options for data modification, deletion, and revocation of consent should be provided. During the data sharing process, a “transparent data use statement” should be made available to ensure that the public has a clearer understanding of the data’s destination, use, and potential impact. Furthermore, the moderating role of delayed discounting on data-sharing decisions offers new perspectives for research. For individuals with a high delayed discounting rate, the immediate risks associated with data sharing may be overemphasized, potentially overshadowing the long-term health benefits. To address this issue, future data-sharing initiatives could focus on increasing public awareness of the long-term benefits of data sharing, emphasizing its potential in health management, disease prevention, and medical research. This approach could help individuals balance short-term privacy protection with long-term benefits.

### Limitations

There are some limitations in our study. First, we classified health data only into PGD and clinical medical data. Future research could categorize them in more detail. Second, although we included variables such as sex, age, and education in our questionnaire, we did not analyze other personal characteristics, such as history of illness. Additionally, our study did not provide a detailed categorization of the recipients of data sharing. Future studies could specify recipients more precisely, categorizing them as hospitals, insurance agencies, medical device companies, and so on.

### Conclusions

Our study has revealed the multifaceted impacts of different types of health data on public willingness to data sharing and highlighted the significant roles of data acquisition costs, delay discounting rates, and health data control rights in this process. These findings not only contribute to a deeper understanding of public attitudes and behaviors regarding data sharing but also provide valuable insights for the development of effective data-sharing policies and privacy protection measures.

## Abbreviations

HIE: health information exchange
HIPAA: Health Insurance Portability and Accountability Act
OR: odds ratio
PCD: patient-centered design
PGD: patient-generated data
